# Outcome of Critically Ill COVID-19 Patients According to the Setting of Corticosteroid Initiation—A Retrospective Observational Cohort Study

**DOI:** 10.3390/jpm11121359

**Published:** 2021-12-13

**Authors:** Sebastian Voicu, Thomas Lacoste-Palasset, Isabelle Malissin, Shana Bekhit, Eléonore Cauchois, Sirine Dahmani, Melkir Saib, Caroline Grant, Giulia Naim, Aymen M’Rad, Adrien Pepin-Lehaleur, Jean-Michel Ekhérian, Nicolas Deye, Bruno Mégarbane

**Affiliations:** Department of Medical and Toxicological Critical Care, Lariboisière Hospital, INSERM UMRS-1144, Paris-University, 75010 Paris, France; sebastian.voicu@aphp.fr (S.V.); thomas.lacoste-palasset@aphp.fr (T.L.-P.); isabelle.malissin@aphp.fr (I.M.); shanabekhit@gmail.com (S.B.); eleonore.cauchois@gmail.com (E.C.); dahmani.sirine@hotmail.fr (S.D.); melkirsaib@yahoo.fr (M.S.); caroline.grant@orange.fr (C.G.); giulia.naim@aphp.fr (G.N.); mrad.aymen@gmail.com (A.M.); Adrien.Pepin-lehaleur@aphp.fr (A.P.-L.); jean-michel.ekherian@wanadoo.fr (J.-M.E.); Nicolas.deye@aphp.fr (N.D.)

**Keywords:** corticosteroid, COVID-19, dexamethasone, intensive care unit, mortality, survival

## Abstract

(1) Background: Corticosteroids lower 28-day all-cause mortality in critically ill COVID-19 patients. However, the outcome of COVID-19 patients referred to the intensive care unit (ICU) for respiratory deterioration despite corticosteroids initiated during hospitalization before ICU admission has been poorly investigated. Our objective was to determine survival according to corticosteroid initiation setting. (2) Methods: We conducted a cohort study including all successive critically ill COVID-19 patients treated with corticosteroids and managed in our ICU. We compared survival, whether corticosteroids were initiated before (Cb-group) or after ICU admission (Ca-group), using a propensity score matching. (3) Results: Overall, 228 patients (67 years (56–74); 168M/60F; invasive mechanical ventilation on admission, 17%) were included with 63 patients in the Cb-group and 165 patients in the Ca-group. Survival to hospital discharge was 43% versus 69%, respectively (*p* = 0.001). In a multivariable analysis, factors associated with death were age (odds ratio, 1.07; 95%-confidence interval, (1.04–1.11); *p* < 0.0001), the sequential organ failure assessment (SOFA) score on ICU admission (1.30 (1.14–1.50); *p* = 0.0001) and corticosteroid initiation before ICU admission (2.64 (1.30–5.43); *p* = 0.007). No significant differences in outcome related to corticosteroid regimen were found. (4) Conclusions: Critically ill COVID-19 patients transferred to the ICU with deterioration despite corticosteroids initiated before admission have a less favorable outcome than patients receiving corticosteroids initiated after ICU admission.

## 1. Introduction

Pneumonia attributed to coronavirus disease-2019 (COVID-19) is still a major issue worldwide despite all efforts to understand its pathophysiology and various repurposed and innovative therapies tested to improve survival. In vulnerable patients, COVID-19 may lead to life-threatening hypoxemia requiring respiratory support including invasive mechanical ventilation. Despite all improvements in COVID-19 management in the last 18 months, critically ill patients still present an elevated mortality rate, generally found to be beyond 30% [1,2].

Inflammation is one of the cornerstones of lung injury pathophysiology in COVID-19-related pneumonia [3]. Immunomodulatory drugs such as corticosteroids and monoclonal anti-interleukin-6 receptor antibodies have been shown to improve survival based on large randomized clinical trials and meta-analyses [4,5,6,7,8,9]. Corticosteroids including dexamethasone reduced 28-day all-cause mortality in COVID-19 pneumonia patients [4], encouraging their systematic administration to patients requiring oxygen as soon as admitted to the medical ward before the onset of respiratory or any other organ failure as well as to patients with severe respiratory failure requiring management in the intensive care unit (ICU). However, some patients on corticosteroids started in the medical ward might deteriorate and be subsequently referred to the ICU for respiratory support. Strikingly, the exact outcome of such patients is still poorly known, whereas due to the increasing corticosteroid use in the medical wards, their prevalence among COVID-19 patients managed in the ICU is increasing. Therefore, we designed an observational study aiming to investigate outcome of critically ill COVID-19 pneumonia patients in relation to the setting of corticosteroid initiation, i.e., before versus after ICU admission. In addition, we aimed to investigate differences in outcome related to the corticosteroid regimen used in the ICU.

## 2. Materials and Methods

### 2.1. Study Design

We conducted a single-center cohort study performed in our university hospital ICU including all successive critically ill COVID-19 pneumonia patients admitted from March 2020 to June 2021 and treated with corticosteroids as immunomodulatory therapy. Patients younger than 18 years, patients not receiving corticosteroids, patients who received corticosteroids for any other reason than COVID-19-related pneumonia, patients who died within 24 h of the initiation of corticosteroids (i.e., considered as moribund), and patients who refused to participate in this observational research were not included.

Diagnosis of severe acute respiratory syndrome coronavirus-2 (SARS-CoV-2) infection relied on positive polymerase chain reaction (Cobas^®^ SARS-CoV-2 Test, Roche, France; sensitivity limit, 40 cycles) using nasopharyngeal swabs, or upper respiratory/bronchial samples. Pulmonary involvement was assessed based on history, physical examination, hypoxemia requiring >6 L/min oxygen, and thoracic computed tomography-scan if available.

The study was conducted according to the 2013 Declaration of Helsinki of the World Medical Association, undertaken as part of the COVID-ICU and French COVID-19 cohort registries and approved by the ethics committee of our institution (N°, IDRCB, 2020-A00256-33; CPP, 11-20-20.02.04.68737). In accordance with the ethical standards of French legislation, informed consent was waived due to the non-interventional study design that did not modify existing diagnostic or therapeutic strategies. Only the non-opposition of the patient or his legal representative to this observational study was collected.

### 2.2. Patient Management in the ICU and Corticosteroid Treatment

COVID-19 pneumonia patients were admitted to the ICU if requiring >6 L/min oxygen by nasal cannula to maintain SpO_2_ ≥ 94%. Once transferred to the ICU and based on the severity of hypoxemia, they received >6L/min nasal oxygen, high-flow oxygen delivered by specific cannula [10], and non-invasive or invasive mechanical ventilation to treat acute respiratory distress syndrome (ARDS) according to the international guidelines [11,12,13].

Before July 2020, dexamethasone was administered once partial pressure of arterial dioxygen over fraction of inspired dioxygen (PaO_2_/FiO_2_) ratio < 200 mmHg was reached despite optimized mechanical ventilation [11], using 20 mg/day for 5 days followed by 10 mg/day for 5 days (D20/10-regimen) as recommended to treat ARDS [14]. Later, during the pandemic, dexamethasone 6 mg/day for 10 days (D6-regimen) was suggested as lifesaving in COVID-19 pneumonia patients based on the RECOVERY study [4]. As soon as the preliminary results were published in July 2020, all COVID-19 patients requiring oxygen were treated with corticosteroids in the medical ward using this dose regimen. If their respiratory conditions continued to deteriorate, patients were transferred subsequently to the ICU where dexamethasone was continued. Once hospitalized in the ICU, both the D6 and D20/10 dose regimens were used according to the physicians in charge, whether dexamethasone was started or continued in the ICU. If required (i.e., insufficient respiratory improvement based on the opinion of the physicians in charge), the duration of corticosteroid could be prolonged up to 20 days in total using the last prescribed dose of each regimen.

According to the advances in knowledge about COVID-19 management and the findings of clinical trials, patients received additional immunomodulatory therapies such as hydroxychloroquine/azithromycin and lopinavir/ritonavir combinations [15,16] at the beginning of the pandemic (March–April 2020) or anakinra (interleukin-1 receptor antagonist) and tocilizumab (interleukin-6 receptor antagonist) [7,8,9] later (especially from March 2021).

### 2.3. Study Groups and Endpoint Definitions

We defined two groups according to the setting of corticosteroid initiation, i.e., patients in whom corticosteroids were initiated in the medical ward before ICU transfer (Cb-group) and patients in whom corticosteroids were initiated once admitted to the ICU (Ca-group).

The main study endpoint was survival to hospital discharge. The additional study endpoint was the number of days alive free of invasive mechanical ventilation at day 28. We aimed to compare these endpoints between the Ca- and Cb-group patients (as main objective) and between patients receiving the D6- and D20/10-regimens (as secondary objective).

### 2.4. Data Collection

The main demographic, clinical, biological, therapeutic and outcome data were collected. The dates of first symptoms, hospital and ICU admissions, and corticosteroid therapy start were recorded. The Sequential Organ Failure Assessment (SOFA) score [17,18] was calculated on ICU admission.

### 2.5. Statistical Analysis

Categorical variables are expressed as frequency (percentage) and continuous variables as median (interquartile range). Comparisons between groups were performed using Chi-2 and Mann-Whitney tests, as appropriate. A multivariable model was built to determine independent factors associated with mortality to hospital discharge including the corticosteroid group (Ca- versus Cb-group) and clinically relevant a priori identified patient characteristics (i.e., age, SOFA score on ICU admission, necessity of invasive mechanical ventilation on admission) previously shown to influence COVID-19 patient outcome [1,2,18], and therapies other than corticosteroids (e.g., tocilizumab and hydroxychloroquine/azithromycin combination) that may have influenced mortality [8,9,10,14,15,16]. A 1:1 propensity score matching between patients from the Cb- and Ca-groups was performed using the nearest neighbor method without replacement with a caliper of width equal to 0.1 [19] to assess the impact of respiratory deterioration requiring ICU admission in COVID-19 patients already treated with corticosteroids. The propensity score was calculated using a logistic regression including age, gender, PaO_2_/FiO_2_ ratio, SOFA score on admission, invasive mechanical ventilation on admission and tocilizumab therapy, to allow comparability of the two groups. The quality of matching was assessed using the standardized mean difference. The odds ratios (OR) and their 95% confidence intervals were calculated. Statistical analyses were performed using the software R-3.6.1 for Windows^®^ (R Foundation for Statistical Computing, Vienna Austria; https://www.r-project.org/, accessed on 5 December 2021). *p*-values ≤0.05 were considered as significant.

## 3. Results

### 3.1. Patient Characteristics

**Figure 1:**
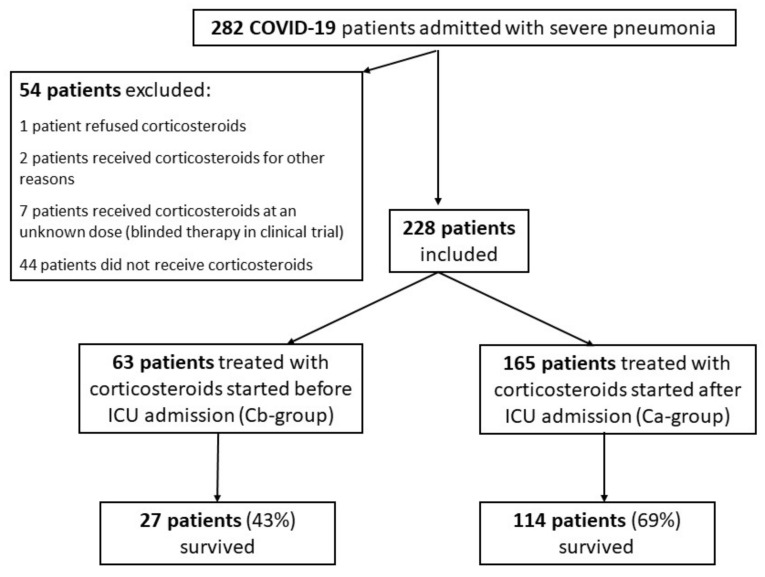
Flow chart of the study.

During the 15-month study period, 869 COVID-19 patients were managed in our institution, including 282 critically ill patients (32%) admitted to our ICU with severe pneumonia (Figure 1). Hypoxemia defined as >6 L/min requirement was the sole criterion of ICU admission of COVID-19 pneumonia patients. Fifty-four patients were not included in the study, including 44 patients who did not receive corticosteroids during the first wave (March–April 2021), as their benefit was uncertain. Therefore, 228 patients (age, 67 years (56–74); 168M/60F; SOFA score on ICU admission, 4 (2–5); and invasive mechanical ventilation on ICU admission, 39/228 (17%)) were included in the study. Percentage of COVID-19 lung injuries evaluated by computed tomography-scan, 48 h before or after ICU admission, was 50% (40–50%). Sixty-three patients were included in the Cb-group and 165 patients in the Ca-group. Characteristics of the patients are shown in Table 1. The overall rate of survival to hospital discharge was 62% and the number of days free of invasive mechanical ventilation was 7 days (0–28).

The Cb-group patients represented 18% (20/109) of the COVID-19 patients admitted to the ICU in 2020 and 36% (43/119) of those admitted in 2021 (*p* = 0.003). Ten patients in this group initially received corticosteroids other than dexamethasone (i.e., prednisolone in 4 patients and methylprednisolone in 6 patients) and treatment was changed to dexamethasone on ICU admission. The equivalent dose of dexamethasone administered to these 10 patients in the medical ward was 14 mg/day (12–33).

### 3.2. Comparisons According to the Corticosteroid Initiation Setting

**Figure 2:**
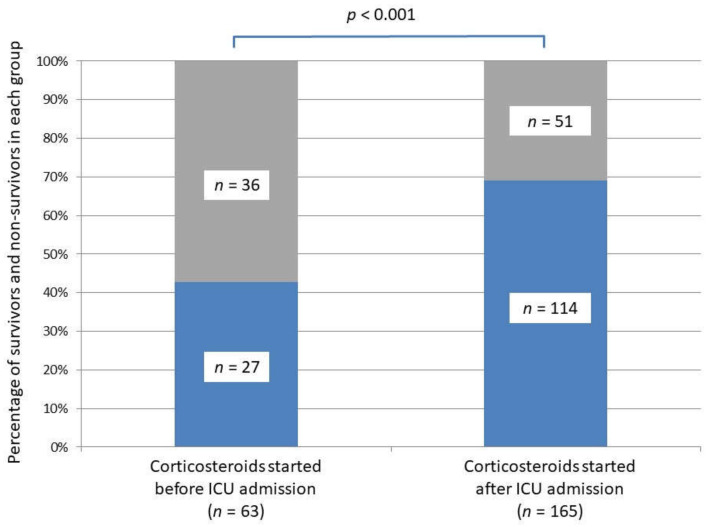
Survival to hospital discharge in the two critically ill COVID-19 groups according to the corticosteroid initiation setting, i.e., before versus after intensive care unit admission. Survivors are represented in blue, non-survivors in grey.

Based on univariate analyses, survival to hospital discharge was lower in the Cb-group compared to the Ca-group (43% versus 69%, *p* < 0.001) (Table 1; Figure 2). The number of days free of invasive mechanical ventilation was lower in the Cb-group compared to the Ca group (7 days (0–28) versus 28 days (2–28), *p* = 0.03).

### 3.3. Prognostic Factors in the Critically Ill Patients Treated with Corticosteroids

Based on a multivariable analysis, factors independently associated with mortality to hospital discharge were age (OR, 1.07 (1.04–1.11); *p* < 0.0001), SOFA score on ICU admission (OR, 1.30 (1.14–1.50); *p* = 0.0001) and corticosteroids started before ICU admission (OR, 2.64 (1.30–5.43), *p* = 0.007) (Table 2).

### 3.4. Propensity Score

Fifty-three patients from the Cb-group were matched with 53 patients from the Ca-group. Matching on age, gender, PaO_2_/FiO_2_ ratio, SOFA score on admission, invasive mechanical ventilation on admission and tocilizumab therapy was well balanced with a standardized mean difference of 0.2 or less (Table 3). After propensity score matching, survival to hospital discharge was lower in the Cb- compared to the Ca-group (45% versus 68%, *p* = 0.03) whereas no significant difference existed regarding the number of days alive free of mechanical ventilation at day 28 (9 days (0–28) versus 28 days (2–28), *p* = 0.11) between these two groups.

### 3.5. Role of the Corticosteroid Dose Regimen and Subgroup Analyses

Once admitted to the ICU, 112 patients were treated with the D6 regimen (33 patients of the Cb-group (52%) versus 79 patients of the Ca-group (48%)) whereas 116 patients were treated with the D20/10 regimen (30 patients of the Cb-group (48%) versus 86 patients of the Ca-group (52%)) (See the Supplementary Table S1). Survival rate and number of days free of invasive mechanical ventilation did not significantly differ according to the dexamethasone regimen.

In the Cb-group, non-survivors presented higher SOFA scores (4 (3–5) versus 3 (2–4), *p* = 0.02), higher serum interleukin-6 concentrations (64.0 pg/mL (46.5–267.0) versus 26.6 pg/mL (9.0–111.0), *p* = 0.01) and required more frequently invasive mechanical ventilation on ICU admission (31% versus 7%, *p* = 0.03) than survivors (Table 4).

## 4. Discussion

The most important finding of our study is that critically ill COVID-19 patients treated with corticosteroids initiated in the medical ward and then referred to the ICU for respiratory deterioration have less favorable outcome than patients in whom corticosteroids are initiated after ICU admission.

As corticosteroids became a standard of care in COVID-19 patients with pneumonia requiring oxygen in the medical ward [4,5,6], the proportion of patients transferred to the ICU for respiratory deterioration despite corticosteroid initiation increased with time representing 36% in 2020 versus 18% in 2021 of COVID-19 patients managed in our ICU. Respiratory deterioration despite corticosteroids initiated earlier in the medical ward corresponded to no or insufficient clinical response to this key-immunomodulatory therapy given to prevent life-threatening respiratory failure, therefore explaining the more unfavorable outcome found in the Cb-group patients. Besides older age and higher SOFA score, previously recognized as predictive factors of worse prognosis [1,2,18], our results suggested that poor initial response to corticosteroids in the medical ward requiring secondary ICU referral should be considered as an independent factor of poor prognosis. We thus strongly believe that patients whose respiratory conditions worsen despite corticosteroids should be the focus of future research to identify new effective therapeutic options that are lacking to date to improve outcome more effectively.

High SOFA score, elevated serum interleukin-6 concentration and need for tracheal intubation during the ICU admission day were factors associated with poor survival in patients admitted to the ICU after initiation of corticosteroids in the medical ward. Elevated serum interleukin-6 concentrations on ICU admission suggested a stronger pro-inflammatory response in non-survivors than in survivors. Therefore, our findings suggested that higher corticosteroid doses [20,21,22] or new inflammation-modulating drugs [23,24,25,26] should be used to manage severe COVID-19 pneumonia patients who could be considered as non-respondent to the usual corticosteroid regimens when requiring secondary ICU transfer from the medical ward.

The exact optimal corticosteroid dose regimen to treat oxygen-dependent COVID-19 patients is still unknown. Authors suggested that higher corticosteroid doses [22] and/or high-dose methylprednisolone boluses [20,21] could be effective as rescue therapy and that duration of treatment may have to be adapted to the disease length and severity, instead of being administered on a one size fits all basis [27]. A recently published randomized control study showed that 12 mg/day compared with 6 mg/day dexamethasone did not result in significantly more days alive without life support at 28 days among patients with COVID-19 and severe hypoxemia [28]. However, as in our observational studies, the authors acknowledged that the trial might have been underpowered.

Survival to hospital discharge in our patients was slightly lower than the 90-day survival reported in a large French cohort (62% versus 69.4%, respectively) [1]. Several explanations could be hypothesized including: (i) the more advanced age in our patients (67 years (56–74) versus 63 years (54–71)); (ii) referral to our center of very severe patients requiring vvECMO on admission [29,30,31]; and (iii) patients still hospitalized at the time of vital status assessment on day 90, some of whom might have eventually expired later.

Our study has limitations. The most important one is its small size precluding a multivariable analysis of factors associated with survival in the Cb-group. Therefore, due to its single-center design, generalizability of our findings should be considered once further confirmatory studies are performed. A multicenter study would have increased the study power but might also have introduced heterogeneity in patient management and the type of corticosteroid protocol used. Our study was probably underpowered to detect a difference between the two dexamethasone dose regimens, requiring a larger cohort given the observed survival rates to hospital discharge. Moreover, patients who received the D20/10 regimen tended to be more severe with lower PaO_2_/FiO_2_ ratios (*p* = 0.008) and more often implemented vvECMO on ICU admission (*p* = 0.003) than patients who received the D6 regimen (See Supplementary Table S1), making comparison of survival difficult to undertake with the limited sample size and thus requiring future larger randomized studies. Finally, although the finding that non-survivors in the Cb-group present with higher interleukin-6 concentrations on ICU admission suggests that the inflammatory response may require strengthened immunomodulatory treatment to improve prognosis, our study could not suggest a strategy to achieve this goal but indicates the need for future innovative therapeutic strategies.

## 5. Conclusions

Patients referred to the ICU for respiratory deterioration after corticosteroid initiation in the medical ward present a higher risk of in-hospital death than patients in whom corticosteroid therapy was initiated after ICU admission. Efforts to identify new therapeutic options are still awaited to improve prognosis of COVID-19 patients with life-threatening pneumonia who respond poorly to corticosteroids.

## Figures and Tables

**Table 1:** Characteristics and outcomes of the 228 critically ill COVID-19 patients according to the setting of corticosteroid therapy initiation, i.e., before (Cb-group) versus after (Ca-group) admission to the intensive care unit.

Variable	Overall (*n* = 228)	Cb-Group (*n* = 63)	Ca-Group (*n* = 165)	*p*-Value
Demographics and Comorbidities				
Age (years)	67 (56–74)	70 (64–78)	64 (53–73)	0.001
Male gender, *n* (%)	168 (74)	50 (79)	118 (72)	0.23
Past hypertension, *n* (%)	119 (52)	38 (60)	81 (49)	0.13
Diabetes mellitus, *n* (%)	84 (37)	29 (46)	55 (33)	0.08
Ischemic heart disease, *n* (%)	26 (11)	9 (14)	17 (10)	0.40
Body-mass index (kg/m^2^)	28.5 (25.4–32.9)	29.2 (26.0–32.0)	28.0 (25.3–33.4)	0.69
Tobacco smoking, *n* (%)	16 (7)	2 (3)	14 (9)	0.16
Parameters on ICU Admission				
SARS-CoV-2 (original strain, and alpha, beta, delta, and undetermined *** variants), *n* (%)	189 (83)/26 (11)/8 (4)/2 (1)/3 (1)	50 (79)/10 (16)/1 (2)/1 (2)/1 (2)	139 (84)/16 (10)/7 (4)/1 (1)/2 (1)	0.33
SOFA score *	4 (2–5)	4 (3–5)	4 (2–6)	0.50
Lung injuries by CT-scan (%)	50 (40–60)	50 (30–60)	50 (40–60)	0.77
Blood lactate (mmol/L) *	1.3 (1.0–1.7)	1.5 (1.2–1.9)	1.3 (1.0–1.7)	0.08
PaO_2_/FiO_2_ ratio (mmHg) *	111 (78–171)	93.6 (69–123)	120 (83–180)	0.003
Serum C-reactive protein (mg/L) *	140 (78–220)	99 (43–156)	160 (95–232)	<0.001
Serum procalcitonin (ng/mL) *	0.26 (0.12–0.75)	0.18 (0.08–0.42)	0.30 (0.13–0.82)	0.01
White blood cells (G/L) *	8.5 (6.2–11.2)	9.9 (7.9–12.5)	8.2 (5.8–10.5)	<0.001
Peripheral lymphocytes (G/L) *	0.8 (0.5–1.0)	0.6 (0.4–0.9)	0.8 (0.6–1.1)	0.005
Peripheral neutrophils (G/L) *	7.0 (5.0–9.5)	8.6 (6.7–10.4)	6.3 (4.7–9.0)	<0.001
Serum interleukin-6 concentration (pg/mL) *	63.4 (20.6–150.1)	62.1 (20.5–141.7)	67.0 (20.8–162.2)	0.62
Invasive mechanical ventilation on ICU admission day *	39 (17)	13 (21)	26 (16)	0.38
Vasopressors, *n* (%) *	26 (11)	3 (5)	23 (14)	0.051
vvECMO, *n* (%) *	13 (6)	2 (3)	11 (7)	0.31
Time from hospital to ICU admission (days)	1 (0–4)	5 (3–10)	0 (0–2)	<0.001
Corticosteroid Treatment and Additional Therapies in the ICU **				
Time from symptoms to corticosteroids (days)	7 (5–10)	7 (4–9)	8 (6–11)	<0.001
Dexamethasone in the ICU, *n* (%) D6 regimen D20/10 regimen	112 (49) 116 (51)	33 (52) 30 (48)	79 (48) 86 (52)	0.54
Total duration of corticosteroid treatment (days)	10 (10–14)	14 (10–20)	10 (10–10)	<0.001
Tocilizumab, *n* (%)	79 (35)	22 (35)	57 (35)	0.96
Hydroxychloroquine/azithromycin combination, *n* (%)	35 (15)	1 (2)	34 (21)	<0.001
Lopinavir/ritonavir, *n* (%)	6 (3)	0 (0)	6 (4)	0.19
Anakinra, *n* (%)	3 (1)	0 (0)	3 (2)	0.56
Invasive mechanical ventilation, *n* (%)	111 (49)	36 (57)	75 (46)	0.11
Prone positioning, *n* (%)	99 (49)	31 (59)	68 (45)	0.09
Number of proning sessions	3 (1–5)	2 (1–5)	3 (1–4)	0.74
Nitrogen oxide, *n* (%)	47 (21)	16 (26)	31 (19)	0.28
vvECMO, *n* (%)	31 (14)	11 (18)	20 (12)	0.29
Renal replacement therapy, *n* (%)	42 (19)	14 (23)	28 (17)	0.34
ICU Complications and Outcome				
Hospital-acquired infection, *n* (%)	100 (44)	35 (56)	65 (39)	0.03
Number of hospital-acquired infection episodes	2 (1–3)	2 (1–4)	2 (1-2)	0.03
Number of days alive free of mechanical ventilation at day 28 (days)	7 (0–28)	7 (0–28)	28 (2–28)	0.03
Survival to hospital discharge, *n* (%)	141 (62)	27 (43)	114 (69)	<0.001

* Parameters measured on ICU admission. ** No patient received remdesivir. *** Mainly due to extremely low viral load. Dexamethasone dose regimen consisting of 6 mg/day for 10 days (the D6 regimen) or 20 mg/day for 5 days followed by 10 mg/day for 5 days (the D10/20 regimen). CT-scan, computed tomography scan; ICU, intensive care unit; vvECMO, venovenous extracorporeal membrane oxygenation; SOFA, sequential organ failure assessment.

**Table 2:** Multivariable analysis of predictors of in-hospital mortality in 228 critically ill COVID-19 patients with pneumonia treated with corticosteroids.

	OR (CI_95_)	*p*
Corticosteroids started before ICU admission	2.64 (1.30–5.43)	0.007
Invasive mechanical ventilation on ICU admission	2.53 (0.93–7.25)	0.07
Tocilizumab	1.49 (0.76–2.95)	0.25
SOFA on ICU admission *	1.30 (1.14–1.50)	0.0001
Age *	1.07 (1.04–1.11)	<0.0001
Hydroxychloroquine/azithromycin combination	0.67 (0.22–1.86)	0.45

* Per one unit increased. OR, odds ratio; CI_95_, 95% confidence interval; SOFA, sequential organ failure assessment.

**Table 3:** Patient characteristics and outcomes after propensity score matching according to the setting of corticosteroid therapy initiation, i.e., before (Cb-group) versus after (Ca-group) admission to the intensive care unit.

	Cb-Group (*n* = 53)	Ca-Group (*n* = 53)	*p*	SMD
Demographics and Comorbidities				
Age (years)	70 (63–75)	69 (58–77)	0.76	0.12
Male gender, *n* (%)	40 (76)	39 (74)	1	0.04
Past hypertension, *n* (%)	35 (66)	30 (57)	0.43	0.20
Diabetes mellitus, *n* (%)	27 (51)	15 (28)	0.028	0.48
Ischemic heart disease, *n* (%)	9 (17)	7 (13)	0.79	0.11
Body mass index (kg/m^2^)	29.2 (26.2–32.0)	26.7 (23.5–33.4)	0.30	0.11
Tobacco smoking, *n* (%)	2 (4)	3 (6)	1	0.09
Parameters on ICU Admission				
SOFA score *	4 (3–5)	3 (2–5)	0.59	0.06
Blood lactate (mmol/L) *	1.5 (1.2–1.8)	1.3 (1.0–2.1)	0.83	0.07
PaO_2_/FiO_2_ ratio (mmHg) *	94 (70–143)	103 (76–146)	0.61	0.12
Serum C-reactive protein (mg/L) *	102 (47–157)	160 (97–240)	0.002	0.58
Serum procalcitonin (ng/mL) *	0.19 (0.08, 0.61)	0.30 (0.13, 0.64)	0.24	0.21
White blood cells (G/L) *	10.1 (7.9–12.2)	7.6 (5.6–10.0)	0.001	0.64
Peripheral lymphocytes (G/L) *	0.6 (0.4–0.9)	0.8 (0.6–1.0)	0.012	0.50
Peripheral neutrophils (G/L) *	9.0 (6.7–10.4)	6.3 (4.4–8.8)	<0.001	0.70
Serum interleukin-6 (pg/mL)*	54 (13–129)	54 (19–193)	0.40	0.09
Invasive mechanical ventilation on ICU admission day, *n* (%) *	8 (15)	10 (19)	0.80	0.10
Vasopressors, *n* (%) *	2 (4)	5 (9)	0.44	0.23
vvECMO, *n* (%) *	2 (4)	0 (0)	0.50	0.28
Time from hospital to ICU admission (days)	5 (2–10)	1 (0–2)	<0.001	0.70
Corticosteroid Treatment and Additional Therapies in the ICU				
Time from symptoms to corticosteroids (days)	6 (3–9)	8 (6–10)	0.004	0.63
Dexamethasone in the ICU, *n* (%) D6 regimen D20/10 regimen	28 (53) 25 (47)	29 (55) 24 (44)	1	0.04
Corticosteroid treatment duration (days)	14 (10–21)	10 (10–13)	<0.001	0.54
Tocilizumab, *n* (%)	16 (30)	20 (38)	0.54	0.16
Hydroxychloroquine/azithromycin combination, *n* (%)	1 (2)	8 (15)	0.031	0.49
Invasive mechanical ventilation, *n* (%)	29 (55)	20 (38)	0.12	0.35
Prone positioning, *n* (%)	25 (58)	22 (47)	0.30	0.23
Number of proning sessions	2.5 (2.0-5.3)	2.5 (1.0–5.0)	0.54	0.05
Nitrogen oxide, *n* (%)	12 (23)	6 (12)	0.20	0.30
vvECMO, *n* (%)	9 (17)	1 (2)	0.016	0.54
Renal replacement therapy, *n* (%)	13 (25)	4 (8)	0.018	0.49
ICU Complications and Outcome				
Hospital-acquired infection, *n* (%)	29 (55)	19 (36)	0.08	0.39
Number of hospital-acquired infection episodes	2 (1–4)	2 (1, 2)	0.032	0.81
Number of days alive free of mechanical ventilation at day 28 (days)	9 (0–28)	28 (2–28)	0.11	0.35
Survival to hospital discharge, *n* (%)	24 (45)	36 (68)	0.031	0.47

* Parameters measured on ICU admission. Dexamethasone dose regimen consisting of 6 mg/day for 10 days (the D6 regimen) or 20 mg/day for 5 days followed by 10 mg/day for 5 days (the D10/20 regimen). ICU, intensive care unit; vvECMO, venovenous extracorporeal membrane oxygenation; SMD, standardized mean difference; SOFA, sequential organ failure assessment.

**Table 4:** Comparisons between survivors and non-survivors among the 63 critically ill patients treated with corticosteroids before admission to the intensive care unit (Cb-group patients).

	Overall (*n* = 63)	Non-Survivors (*n* = 36)	Survivors (*n* = 27)	*p*-Value
Demographics and Comorbidities				
Age (years)	70 (64–78)	73 (66–79)	69 (62–74)	0.13
Male gender, *n* (%)	50 (79)	28 (78)	22 (81)	0.72
Past hypertension, *n* (%)	38 (60)	23 (64)	15 (56)	0.50
Diabetes mellitus, *n* (%)	29 (46)	18 (50)	11 (41)	0.47
Ischemic heart disease, *n* (%)	9 (14)	7 (19)	2 (7)	0.28
Body mass index (kg/m^2^)	29.2 (26.0–32.0)	29.6 (27.3–32.0)	27.8 (24.1–31.7)	0.29
Tobacco smoking, *n* (%)	2 (3)	1 (3)	1 (4)	1.0
Parameters on ICU Admission				
SOFA score *	4 (3–5)	4 (3–5)	3 (2–4)	0.02
PaO_2_/FiO_2_ ratio (mmHg) *	94 (69–122)	81 (69–127)	98 (71–121)	0.42
Blood lactate (mmol/L) *	1.50 (1.20–1.90)	1.50 (1.20–1.85)	1.50 (1.15–1.90)	0.75
Serum C-reactive protein (mg/L) *	99 (43–156)	85 (39–144)	130 (47–176)	0.52
Serum procalcitonin (ng/mL) *	0.18 (0.08–0.42)	0.21 (0.09–0.41)	0.18 (0.08–0.38)	0.52
White blood cells (G/L) *	9.9 (7.9–12.5)	9.5 (7.2–14.0)	10.5 (8.5–11.8)	0.60
Peripheral lymphocytes (G/L) *	0.6 (0.4–0.9)	0.6 (0.4–0.9)	0.6 (0.4–0.9)	0.46
Peripheral neutrophils (G/L) *	8.6 (6.7–10.4)	8.2 (5.9–11.9)	9.1 (7.6–10.1)	0.72
Serum interleukin-6 (pg/mL) *	62.2 (20.5–142.0)	64.0 (46.5–267.0)	26.6 (9.0–111.0)	0.01
Invasive mechanical ventilation on ICU admission day, *n* (%) *	13 (21)	11 (31)	2 (7)	0.03
Vasopressors, *n* (%) *	3 (5)	3 (15)	0 (0)	0.12
vvECMO, *n* (%) *	2 (3)	2 (6)	0 (0)	0.50
Time from hospital to ICU admission (days)	5 (2.5–10)	6 (3–16)	4 (2–9)	0.04
Corticosteroid Treatment and Additional Therapies in the ICU				
Time from symptoms to corticosteroids (days)	7 (4–9)	6 (4–9)	7 (5–9)	0.84
Corticosteroid before ICU, *n* (%) D6 regimen Other corticosteroid regimen	53 (84) 10 (16)	31 (86) 5 (14)	22 (81) 5 (19)	0.73
Dexamethasone in the ICU, *n* (%) D6 regimen D20/10 regimen	33 (52) 30 (48)	21 (58) 16 (44)	12 (44) 14 (54)	0.56
Corticosteroid treatment duration (days)	14 (10–20)	13 (10–23)	15 (11–20)	0.39
Tocilizumab, *n* (%)	22 (35)	13 (36)	9 (33)	0.82
Hydroxychloroquine/azithromycin combination, *n* (%)	1 (2)	1 (3)	0 (0)	1.0
Time from corticosteroid initiation to ICU transfer (days)	5 (2–9)	4 (2–8)	5 (3–9)	0.83
Invasive mechanical ventilation, *n* (%)	36 (57.1)	30 (83)	6 (22)	<0.001
Prone positioning, *n* (%)	31 (59)	24 (75)	7 (33)	0.004
Number of proning sessions	2 (1–5)	2 (1–6)	3 (1–5)	0.78
Nitrogen oxide, *n* (%)	16 (26)	16 (44)	0 (0)	<0.001
vvECMO, *n* (%)	11 (18)	11 (31)	0 (0)	0.001
Renal replacement therapy, *n* (%)	14 (23)	11 (31)	3 (12)	0.12
ICU Complications and Outcome				
Hospital-acquired infection, *n* (%)	35 (56)	27 (75)	8 (30)	0.001
Number of hospital-acquired infection episodes	2 (1–4)	2 (2-3)	4 (1–4)	0.53

* Parameters measured at intensive care unit admission. Dexamethasone dose regimen consisting of 6 mg/day for 10 days (the D6 regimen) or 20 mg/day for 5 days followed by 10 mg/day for 5 days (the D10/20 regimen). ICU, intensive care unit; vvECMO, venovenous extracorporeal membrane oxygenation; SOFA, sequential organ failure assessment.

## Data Availability

Bruno Mégarbane has full access to all data and takes responsibility for the data integrity and its analysis accuracy. Data supporting reported results can be obtained from the corresponding author if reasonably justified.

## Supplementary Materials

The following are available online at https://www.mdpi.com/article/10.3390/jpm11121359/s1, Table S1: Characteristics and outcomes of 228 critically ill COVID-19 patients managed with corticosteroid therapy according to the dose regimen used.
