# Supplementary material for: Outcome of Critically Ill COVID-19 Patients According to the Setting of Corticosteroid Initiation—A Retrospective Observational Cohort Study
Source: J Pers Med. 2021 Dec 13;11(12):1359. doi: 10.3390/jpm11121359 (PMC8706060; doi:10.3390/jpm11121359)
Supplement: Supplementary file 1 [file jpm-11-01359-s001.zip › jpm-1455089-supplementary.pdf]

Table S1. Characteristics and outcomes of 228 critically ill COVID-19 patients managed with corticosteroid therapy according to the dose regimen used.

Variable	Overall (*n* = 228)	D6 regimen (*n* = 112)	D20/10 regimen (*n* = 116)	*p*-Value
**Demographics and Comorbidities**				
Age (years)	67 (56–74)	69 (57–77)	64 (56–73)	0.04
Male gender, *n* (%)	168 (74)	85 (76)	83 (72)	0.55
Past hypertension, *n* (%)	119 (52)	61 (55)	58 (50)	0.51
Diabetes mellitus, *n* (%)	84 (37)	42 (38)	42 (36)	0.89
Ischemic heart disease, *n* (%)	26 (11)	10 (9)	16 (14)	0.30
Body-mass index (kg/m^2)	28.5 (25.4–32.9)	27.9 (24.4–32.0)	28.9 (26.1–34.1)	0.02
Tobacco smoking, n (%)	16 (7)	4 (4)	12 (10)	0.07
**Parameters on ICU Admission**				
SOFA score *	4 (2-5)	4 (3-5)	4 (2–6)	0.44
Blood lactate (mmol/L) *	1.3 (1.0–1.7)	1.3 (1.0–1.7)	1.3 (1.0–1.8)	0.81
PaO_2/FiO_2 ratio (mmHg) *	111 (78–171)	135 (87–188)	97 (75–148)	0.008
Serum C-reactive protein (mg/L) *	140 (78–220)	151 (82–224)	134 (73–207)	0.24
Serum procalcitonin (ng/mL) *	0.26 (0.12–0.75)	0.30 (0.14–1.18)	0.21 (0.11–0.56)	0.06
White blood cells (G/L) *	8.5 (6.2–11.2)	8.4 (6.1–10.7)	8.7 (6.2–11.5)	0.48
Peripheral lymphocytes (G/L) *	0.8 (0.5–1.0)	0.7 (0.5–1.0)	0.8 (0.6–1.1)	0.15
Peripheral neutrophils (G/L) *	7.0 (5.0–9.5)	6.8 (5.0–9.5)	7.3 (5.0–9.5)	0.96
Serum interleukin-6 concentration (pg/mL) *	63.4 (20.6–150.1)	59.1 (27.2–141.1)	65.8(19.0–161.1)	0.95
Invasive mechanical ventilation on ICU admission day *	39 (17)	16 (14)	23 (20)	0.30
Vasopressors, *n* (%) *	26 (11)	9 (8)	17 (15)	0.15
vvECMO, *n* (%) *	13 (6)	1 (1)	12 (10)	0.003
Time from hospital to ICU admission (days)	1 (0-4)	1 (0-5)	1 (0-4)	0.69
**Corticosteroid Treatment and Additional Therapies in the ICU**				
Time from symptoms to corticosteroids (days)	7 (5–10)	7 (5–10)	8 (5–11)	0.24
Corticosteroid initiation setting, *n* (%)				
before ICU admission (Cb-group)	63 (28)	33 (30)	30 (26)	0.54
after ICU admission (Ca-group)	165 (72)	79 (70)	86 (74)	
Total duration of corticosteroid treatment (days)	10 (10–14)	10 (10–14)	10 (10–15)	0.27
Tocilizumab, *n* (%)	79 (35)	16 (14)	63 (54)	<0.001
Hydroxychloroquine/azithromycin combination, *n* (%)	35 (15)	0 (0.0)	35 (30)	<0.001
Invasive mechanical ventilation, *n* (%)	111 (49)	48 (43)	63 (54)	0.09
Prone positioning, *n* (%)	99 (49)	42 (45)	57 (51)	0.40
Number of proning sessions	3 (1–5)	2 (1–3)	3 (1–5)	0.40
Nitrogen oxide, *n* (%)	47 (21)	20 (18)	27 (24)	0.33
vvECMO, *n* (%)	31 (14)	11 (10)	20 (17)	0.12
Renal replacement therapy, *n* (%)	42 (19)	22 (20)	20 (17)	0.73
**ICU Complications and Outcome**				
Hospital-acquired infection, *n* (%)	100 (44)	49 (44)	51 (44)	1
Number of hospital-acquired infection episodes	2 (1–3)	2 (1–2)	2 (1–3)	0.35
Number of days alive free of mechanical ventilation at day 28 (days)	7 (0–28)	28 (1–28)	20 (0–28)	0.58
Survival to hospital discharge, *n* (%)	141 (62)	68 (61)	73 (63)	0.79

* Parameters measured at intensive care unit admission. Dexamethasone dose regimen consisting of 6 mg/day for 10 days (the D6 regimen) or 20 mg/day for 5 days followed by 10 mg/day for 5 days (the D10/20 regimen). ICU, intensive care unit; vvECMO, venovenous extracorporeal membrane oxygenation; SOFA, sequential organ failure assessment.
